# Supplementary material for: Content-rich biological network constructed by mining PubMed abstracts
Source: BMC Bioinformatics. 2004 Oct 8;5:147. doi: 10.1186/1471-2105-5-147 (PMC528731; doi:10.1186/1471-2105-5-147)
Supplement: Additional File 2 — The original results of the above study (non-essential files are deleted to keep the file size under the limit set by BMC bioinformatics). [file 1471-2105-5-147-S2.bz2 › chilibotAdditionalFile2/dip10/247ID8978688E810/html/CUT1_CUT2.html]

 


 **CUT1** and **CUT2** 
  
Found 46 abstracts in PubMed, retrieved 10.  
 

 What does Google say? 
 PDF only 
| .edu only 

---

**Interactive relationship** (e.g. stimulation, inhibition, etc)

**Neutral relationship**- A palindrome binding protein PBP previously cloned binds only to palindrome 1 of  **cut1**  promoter but not palindrome 1 of  **cut2**  3 which contains two base substitutions.  Ref: 11756444 J Biol Chem, 2002
- Thus, CTF1 beta is involved in the constitutive expression of  **cut2**  that causes production of low levels of cutin monomers that strongly induce  **cut1**  using CTF1 alpha as the transcription factor.  Ref: 11756444 J Biol Chem, 2002
- Amino acid sequence deduced from the nucleotide sequence of  **cut1**  and  **cut2**  3 matched with that of the peptides from cutinase 1  [ **CUT1** ]  and cutinase  [ **CUT1** ]  2, respectively, isolated from F. solani pisi grown on cutin as the sole carbon source.  Ref: 11756444 J Biol Chem, 2002
- In yeast, CTF1 beta transactivates  **cut2**  promoter but not  **cut1**  promoter unless its palindrome 1 is mutated, unlike CTF1 alpha which transactivates  **cut1** .  Ref: 11756444 J Biol Chem, 2002

**Non-interactive relationship** (e.g. studied together, co-existance, homology, etc.)

- In this review, the roles of two proteins essential for the onset of anaphase in fission yeast,  **Cut2**  securin and  **Cut1**  separin  [ **CUT1** ] , are discussed with regard to cell cycle regulation, and compared with the postulated roles of homologous proteins in other organisms.  Ref: 10651900 Genes Cells, 2000
- We cloned three highly homologous cutinase  [ **CUT1** ]  genes,  **cut1** ,  **cut2** , and cut3, from Fusarium solani f.  Ref: 11756444 J Biol Chem, 2002
- BACKGROUND The fission yeast Schizosaccharomyces pombe separase  **Cut1**  and securin  **Cut2**  are required for anaphase specific activation of proteolysis that leads to proper sister chromatid separation.  Ref: 12653962 Genes Cells, 2003
- ATP binding cassette ABC transporters mediating the uptake of carbohydrates comprise two subfamilies  **CUT1** ,  **CUT2**  that differ with respect to the chemical nature of their substrates, subunit composition, and conserved sequence motifs.  Ref: 11421277 Res Microbiol< MedlineTA>Res Microbiol, 2003
- Fission yeast  **Cut1**  separase resides in interphase cytoplasm and mobilizes to the spindle and the spindle pole bodies SPBs in mitosis, while  **Cut2**  securin remains in the nucleus from interphase to metaphase, and temporarily locates at the short spindle.  Ref: 12390246 Genes Cells, 2002
- roles of  **Cut1**  separin  [ **CUT1** ]  and  **Cut2**  securin.  Ref: 10651900 Genes Cells, 2000
